# Bubble-mediated transport of benthic microorganisms into the water column: Identification of methanotrophs and implication of seepage intensity on transport efficiency

**DOI:** 10.1038/s41598-020-61446-9

**Published:** 2020-03-13

**Authors:** Sebastian F. A. Jordan, Tina Treude, Ira Leifer, René Janßen, Johannes Werner, Heide Schulz-Vogt, Oliver Schmale

**Affiliations:** 1 Leibniz Institute for Baltic Sea Research Warnemünde, Rostock, Germany; 2 University of California, Los Angeles Department of Earth, Planetary, and Space Sciences, Los Angeles, California USA; 3 Bubbleology Research International, Solvang, California USA

**Keywords:** Biogeochemistry, Ocean sciences, Molecular ecology

## Abstract

Benthic microorganisms transported into the water column potentially influence biogeochemical cycles and the pelagic food web structure. In the present study six gas-releasing vent sites in the Coal Oil Point seep field (California) were investigated, and the dislocation of microorganisms from the sediment into the water column via gas bubbles released from the seabed was documented. It was found that the methanotrophs transport efficiency was dependent on the volumetric gas flow, with the highest transport rate of 22.7 × 10^3^ cells mL_gas_^−1^ at a volumetric gas flow of 0.07 mL_gas_ s^−1^, and the lowest rate of 0.2 × 10^3^ cells mL_gas_^−1^ at a gas flow of 2.2 mL_gas_ s^−1^. A simple budget approach showed that this bubble-mediated transport has the potential to maintain a relevant part of the water-column methanotrophs in the seep field. The bubble-mediated link between the benthic and pelagic environment was further supported by genetic analyses, indicating a transportation of methanotrophs of the family *Methylomonaceae* and oil degrading bacteria of the genus *Cycloclasticus* from the sediment into the water column. These findings demonstrate that the bubble-mediated transport of microorganisms influences the pelagic microbial abundance and community composition at gas-releasing seep sites.

## Introduction

The exchange of nutrients and organic matter between benthic and pelagic compartments affects the composition of the respective microbial communities and thus their functional capabilities in shelf seas. Microorganisms transport to and from the sediment and water column also has been described and occurs by a variety of exchange processes. For example, the suspension of benthic bacteria from beach material within the splash zone can facilitate cell transport into coastal water (over-beach transport)^1,2^. In addition, bacteria located at shallow sediment depths can be mobilized by infiltrating seawater and then carried to deeper sediment strata, from where they can be injected into the water column by submarine groundwater discharge (through-beach transport)^3^. Another bentho-pelagic transport mechanism in shallow waters is sediment resuspension triggered by wind-induced mixing, breaking waves, tides, bioturbation, and gas bubble transport^4,5^. Microorganisms in the sediments usually are associated with fine particles (<60 µm)^6^ at concentrations that exceeds those in the water column by up to four orders of magnitude^7^. Resuspension by tidal currents can increase bacteria concentrations especially within the benthic boundary layer, that is thought to be characterized by a distinct microbial community with high activity^8–10^. Together, these exchanges and transport mechanisms may influence the coastal planktonic microbial food web^11–13^ and alter biogeochemical cycles^12,14^.

Shallow marine areas characterized by methane-containing sediments contribute significantly to atmospheric methane input^15,16^. Here, methane migrates from the seabed into the water column via pore water diffusion, advective fluid flow, and/or bubble ebullition^17^. Such gas bubbles also transfer trace gases (e.g. short-chain hydrocarbons or hydrogen sulfide)^18^ from the sediment to the water column together with mineral particles^19^, organic matter^20^, and microorganisms^7,20^. Surfactants attached to the bubbles’ gas/water interfaces can be released by shear stress, bubble dissolution^21^, or bursting at the sea surface^22–24^. Bubble transport is the most efficient pathway of methane transfer between the sediment and atmosphere and bypasses the very effective anaerobic and aerobic microbial methane oxidation^17^. After methane is released from the sediment, several processes determine the amount that reaches the sea surface, especially water depth^18,25^, ocean currents and mixing^26^, water-column stratification^27–30^, and microbial methane oxidation^17,31^. Microbial methane sink mechanisms reduce the annual seabed methane emissions to the atmosphere from 8–65 Tg CH_4_^17^ to ~1–35 Tg CH_4_, respectively^32^.

Methane-oxidizing bacteria (MOB) are frequently found in the water column above methane seep sites^33–36^, but how these slow-growing microorganisms^37^ achieve a significant population density in waters that are renewed continuously by local currents is unclear^7,38^. The findings of previous studies conducted in areas surrounding seep sites^7,29,39,40^ and mud volcanos^35^ suggested that anaerobic methanotrophic archaea and MOB are transported by methane seep bubbles into the water column^35,36^. This hypothesis was based on the higher abundance of MOB in the vicinity of gas bubble flares than in nearby sites not affected by gas bubble emissions from the sediment. In a pilot study at one gas bubble releasing vent site at a shallow nearshore seep field in California, Schmale *et al*.^7^ found first indications of a direct bentho-pelagic transport of MOB, by sampling escaping bubbles directly at the gas-releasing vent hole. A comparison showed that the natural bubbles released from the sediment transported more MOB cells than the bubbles that were released from an engineered gas outlet above the sediment surface. Although the factors controlling bubble-mediated transport were unknown, Schmale *et al*.^7^ hypothesized that transported MOB can inoculate overlaying waters, thereby enhancing the efficiency of the pelagic methane sink.

In this detailed mechanistic study, we conducted a series of bubble-catcher experiments at two different seep sites to assess the influence of the parameters seep intensity, bubble size distribution, and vent density on the efficiency of the bubble-mediated transport of benthic microorganism into the water column. Selected genetic community comparisons of sedimentary, pelagic, and transported microorganisms enabled us to further verify the existence of the Bubble Transport Mechanism and to discuss its impact on biogeochemical transformations in the water column. Finally, a simple budget calculation was applied to assess the impact of the Bubble Transport Mechanism on the water column MOB abundance above the Coal Oil Point (COP) seep field.

## Results

### Distribution of vent sites in the study areas

**Figure 1:**
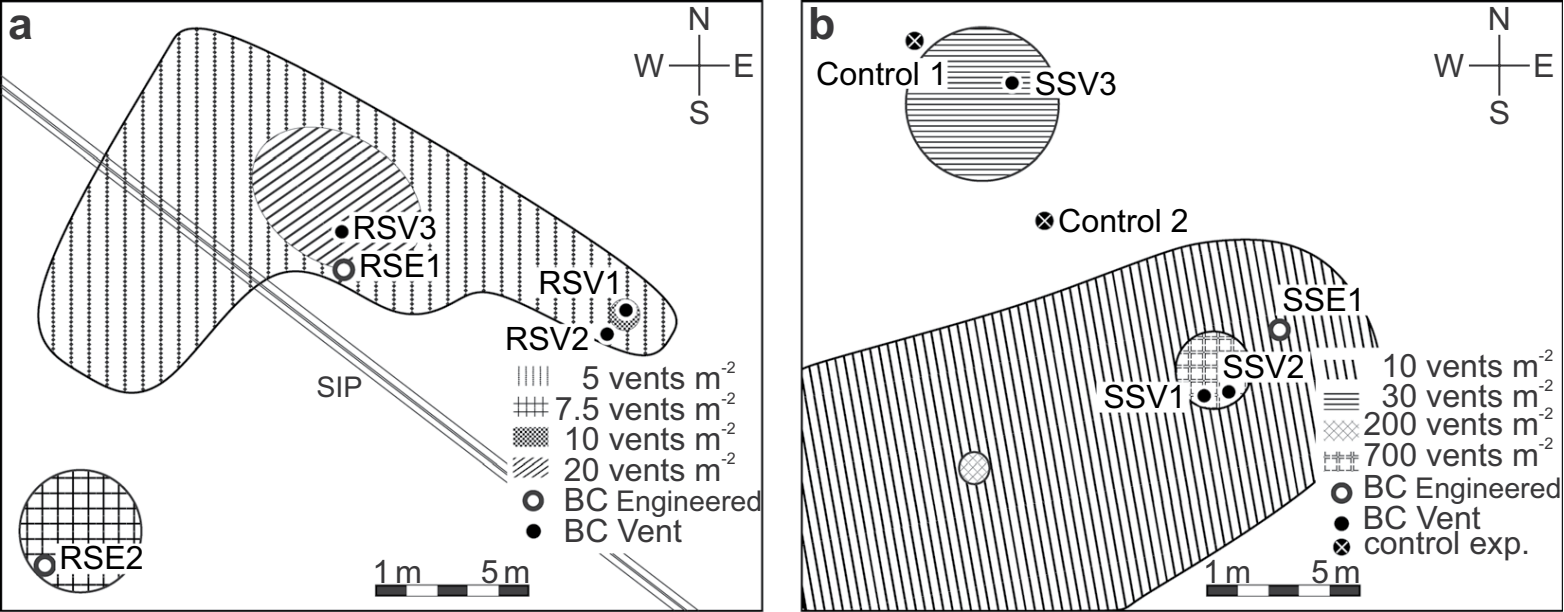
Schematic map of vent distribution and density at (**a**) Rostocker Seep (RS) and (**b**) the Isla Vista Super Seep (SS). Locations of the Bubble Catcher (BC) vent, engineered, and control experiments are marked. The double line illustrated in (**a**) represents the two seawater intake pipes (SIP) that transport seawater from further offshore to the University of California, Santa Barbara campus. CorelDRAW 2018 (v. 20.1.0.708) was used to draw these maps.

The vent densities of Rostocker Seep (RS) ranged from 5 to 20 vents m^−2^, and at IV Super Seep (SS) from 10 to about 700 vents m^−2^ (Fig. 1). The highest vent density, covering an area of ~16 m^2^, was at IV Super Seep. Gas bubble emissions generally were more vigorous at IV Super Seep than at Rostocker Seep.

### Characterization of sediment and water column

**Figure 2:**
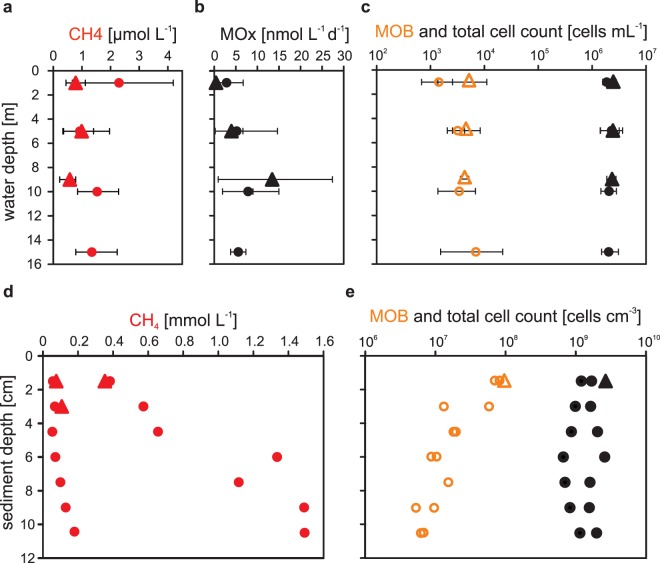
Depth profiles of parameters determined in the water column (**a–c**) and in the sediment (**d,e**) at the Rostocker Seep (triangle) and the IV Super Seep (circles): (**a**) mean water column methane concentration, (**b**) mean water column methane oxidation (MOx), (**c**) abundance of methane-oxidizing bacteria (MOB, open orange symbols) and total cells (black) in the water column, (**d**) sediment methane concentration, (**e**) abundance of MOB (open orange symbols) and total cells (filled black) in the sediment. The bars in (**a-c**) indicate the range of values between three fieldwork days.

The surface sediments at Rostocker Seep and IV Super Seep generally were dominated by a medium sand fraction but the two seeps differed in that IV Super Seep also was characterized by tar deposits in sediment cores and on the sediment surface. Methane sediment concentrations for both seep sites were in the millimolar range and increased with increasing sediment depth, from 0.05 mmol L^−1^ at the sediment surface (0–1.5 cm b.s.f. = centimeters below the seafloor) to 1.5 mmol L^−1^ at 10 cm b.s.f. (Fig. 2d). Total cell counts were consistent over the entire depth range (~1.5 × 10^9^ cells cm^−3^, Supplementary Table S1), although MOB abundances as a percentage of total cell counts decreased from ~7% at the sediment surface (~8 × 10^7^ cells cm^−3^) to ~0.5% at 10.5 cm depth (Fig. 2e).

Water column temperature profiles taken over the course of the sampling campaign indicated the buildup of a thermocline over several hours that then collapsed from mixing during the course of the day (data not shown). Throughout the sampling campaign, bottom currents followed the coast, consistent with typical northern Santa Barbara Basin currents, which are driven by the Davidson Current^41^. Water column turbidity changed on a daily basis, resulting in a visibility that ranged from <0.5 m up to 10 m (reported by divers). The water in the vicinity of both seep sites was enriched in methane (Fig. 2a) which varied on a daily basis. Mean methane concentrations were consistently lower at Rostocker Seep than IV Super Seep by a factor of about two throughout the water column. At Rostocker Seep, methane oxidation rates (MOx) increased with increasing water depth, with the highest rate occurring in the bottom waters (min. 0.1 nmol L^−1^ d^−1^ and max. 27.4 nmol L^−1^ d^−1^, (Fig. 2b). By contrast, at IV Super Seep, the highest oxidation rate was measured in intermediate waters (min. 0.7 nmol L^−1^ d^−1^, max. 15 nmol^−1^ d^−1^, (Fig. 2b). Total cell numbers were consistent throughout the water column and seep sites (~2 × 10^6^ cells mL^−1^, Supplementary Table S2), with MOB abundances of ~4 × 10^3^cells mL^−1^ (0.1–0.2% of DAPI-stained cells) at both seep sites (Fig. 2c). Total cell numbers were approximately three orders of magnitude lower in the water column than in the sediment, and MOB abundances approximately four orders of magnitude lower. Cell-specific methane oxidation rates ranged from 7.4 × 10^−4^ fmol L^−1^ h^−1^ to 1.6 × 10^−2^ fmol L^−1^ h^−1^.

### Bubble size distribution

**Figure 3:**
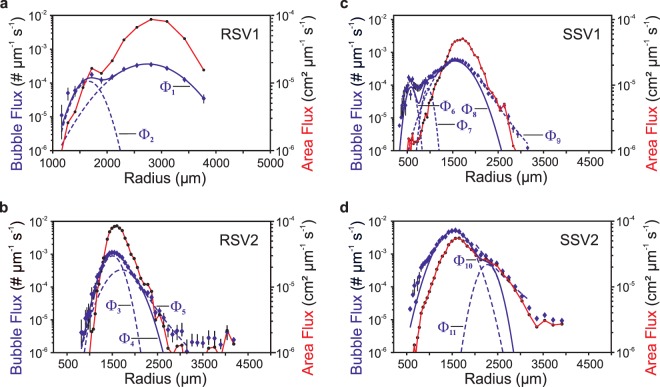
Bubble emission size distribution (Φ) for bubbles crossing an arbitrary height above the seabed per second per unit radius (*r*), for the four Bubble Catcher and BMS studied vents: (**a,b**) Rostocker Seep, (**c,d**) IV Super Seep. Emission modes determined by least-squares linear-regressions, fit equations Φ1–11 can be found in the Supplementary Table S3. Also shown is the bubble surface area flux versus *r*.

A Bubble Measuring System (BMS) collected seep bubble videos for the Rostocker Seep and IV Super Seep where the Bubble Catcher samples were taken. Analysis of the bubble size distribution (the number of bubbles per second per radius increment passing through a plane) for a Rostocker Seep vent revealed two emissions modes at r = 1,660 and 2,750 µm (RSV1, Fig. 3a). This seep was well described by Gaussian functions, i.e., a minor seep with formation not resulting from bubble fragmentation^42^. Emissions were as a bubble pulse of bubbles that lasted ~3.5 s and comprised 774 bubble images. The volumetric gas flow (*Q)* was 1.0 mL_gas_ s^−1^ and the bubble surface area flux (*A*) was 10.9 cm^2^ s^−1^, dominated by the larger mode.

Analysis of 5,236 bubble images from a second, smaller, minor Rostocker Seep vent (RSV2, Fig. 3b) identified a dominant mode at *r* = 1,505 µm and a second much smaller mode at *r* = 1,710 µm. Three complete pulses spanning 20 s were analyzed, with the second pulse lasting ~5 s. Similar to the other pulsing vent at Rostocker Seep, the onset of a bubble pulse at the second vent was accompanied by larger bubbles. *Q* was 0.29 mL_gas_ s^−1^ and *A* was 4.9 cm^2^ s^−1^.

A small minor vent at IV Super Seep (SSV1, Fig. 3c) was analyzed based on 7,850 bubble images and found bubbles spanning 400 < *r* < 8,000 µm with a dominant mode at *r* = 1,540 µm and *Q* = 0.16 mL_gas_ s^−1^, and *A* = 3.0 cm^2^ s^−1^.

The only major vent (where the bubble size distribution is well described by a power law and results from bubble fragmentation) was from the main area of IV Super Seep (SSV2, Fig. 3d); all other vents were minor (Fig. 3a–c). At the IV Super Seep main vent (SSV2), 2,404 bubbles spanning 500–2,600 µm radius were analyzed (Fig. 3d). *Q* was 1.3 mL_gas_ s^−1^ per vent and *A* was 22.7 cm^2^ s^−1^ per vent. *Q* varied by a factor of ~3 on a ~2 s timescale (Supplementary Fig. S1). The dominant emission mode was *r* ~ 1,530 µm, with a second mode at *r* ~ 2,270 µm.

The *A*/*Q* ratios of three of the vents (SSV1, RSV2, SSV2) were similar, ~18 cm^−1^ (Table 1), which was expected given the similarity in the dominant modes of the vents. Emissions were far less steady at the minor than at the major vent and even ceased for ~1 s in the 40 s analyzed video, which also showed clear pulsing. Pulses were associated with larger bubbles. The dominant mode similarity with the major vent indicates that grain size (mid sand fraction at all sampling sites) controlled the emissions and that *Q* was non-critical^43,44^ for all vents. For non-critical flow, *Q* increases the number of bubbles but the size remains the same, for critical flow, the bubble size increases with *Q*. At the minor vent, much less important modes at 580 and 960 µm, corresponding to ~1/3 and 2/3 of the major mode, were detected.

**Table 1:** Overview of the Bubble Catcher experiments. Details of the sampled gas volumes, experiment durations, and volumetric flows of the captured gas are shown.

Experiment	Date	Gas volume [L]	Sampling duration [min]	Volumetric flow, *Q* [mL_gas_ s^−1^]	Transported bubble surface area, *A* [cm^2^ s^−1^]	Ratio bubble surface to volume^−1^, *A/Q* [cm^−1^]
RSV1	01.08.17	0.7	160	0.07	0.8	10.5
RSV2		2.85	160	0.30	5.1	17.1
RSV3	02.08.17	4.85	111	0.73		
RSE1		4.66	67	1.16		
RSE2	15.08.17	4.7	84	0.93		
SSV1	07.08.17	5.13	50	1.71	30.9	18.1
SSV2		5.42	41	2.20	39.6	18.0
SSV3	08.08.17	4.7	197	0.40		
SSE1	14.08.17	5.59	9	10.35		
SSC1	15.09.17	0	67	0		
SSC1		0	69	0		

The BMS data from the IV Super Seep’s major vent showed an upwelling flow (*V*_*up*_) of ~4 cm s^−1^, which agreed well with the field data-derived relationship between *V*_*up*_ and *Q* in Leifer (2010)^45^, in which *V*_*up*_ ~ 4 cm s^−1^ corresponded to *Q* ~ 1.2 mL_gas_ s^−1^. For the IV Super Seep minor vent, *V*_*up*_ ~ 1 cm s^−1^, in good agreement with the *V*_*up*_ of ~ 1.05 cm s^−1^ as reported previously^45^, corresponds to *Q* ~ 1.6 mL_gas_ s^−1^. Interestingly, *V*_*x*_*(r)* for the main vent followed the shape of *V*_*up*_*(r)* for dirty (coated by oil or other surface-active substances) bubbles whereas *V*_*x*_ for the minor vent followed *V*_*up*_*(r)* for clean bubbles.

For Rostocker Seep, *V*_*x*_ suggested clean bubbles. *V*_*up*_ was quite small, roughly 0.25 cm s^−1^ but was challenging to derive for the pulses, as V_x_ varied strongly with time, growing nearly an order of magnitude larger in the middle of the pulse. This introduced a ~30% error into the multiple count correction due to the variation in V_x_ and thus in *Q* and *A*, but not into *A/Q*. As such, there was little change in the *A*/*Q* ratio over time, which allowed its application to the BC volumetric fluxes to derive the BC area fluxes, even though BMS and BC measurements were not concurrent.

### Parametrization of the bubble-mediated transport

**Figure 4:**
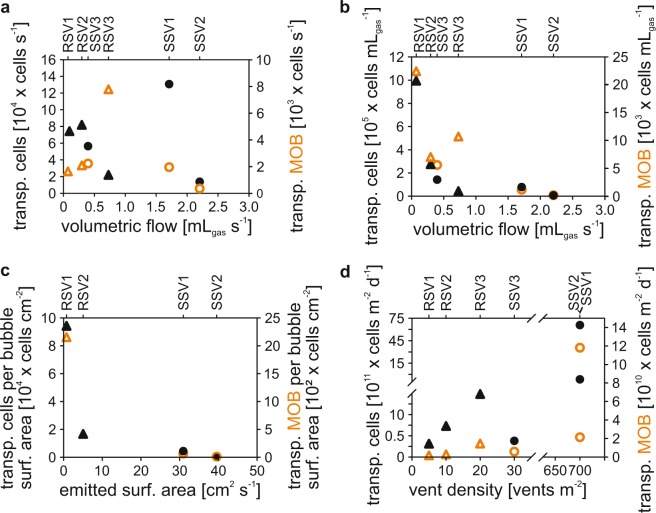
Transported total cells (black) and MOB (orange) at Rostocker Seep (triangle) and IV Super Seep (circles) normalized to (**a**) time and (**b**) emitted gas as a function of volumetric flow, (**c**) bubble surface area as a function of emitted surface area, (d) transported cells per square meter and day versus vent density.

The bubble transport process was studied using the Bubble Catcher (BC) by sampling gas bubbles escaping (1) a natural vent (BC vent) and (2) an engineered gas outlet without any sediment contact (BC engineered). The volumetric flow of the examined vents ranged from 0.07 (±8%) to 0.73 (±2%) mL_gas_ s^−1^ at Rostocker Seep and from 0.04 (±2%) to 2.20 (±3%) mL_gas_ s^−1^ at IV Super Seep (Table 1). The number of transported cells was normalized for time (Fig. 4a), volume of emitted gas (Fig. 4b), transported bubble surface area (Fig. 4c), and m^2^ seabed seepage area (Fig. 4d) to determine the parameters influencing in the bubble-mediated transport of benthic MOB. MOB were transported in all “BC vent” experiments. The total number of transported cells at the individual vents per time ranged from 1.4 × 10^4^ to 1.3 × 10^5^ cells s^−1^. Transported MOB per vent ranged from 4.1 × 10^2^ to 7.8 × 10^3^ cells s^−1^ (Fig. 4a). The number of transported MOB and total cells per milliliter of emitted gas (Fig. 4b) decreased with increasing volumetric flow, following an inverse trend that indicated the transport of a 100 times more MOB (2.27 × 10^4^ cells mL_gas_^−1^) at the lowest volumetric gas flow (0.07 mL_gas_ s^−1^) than at the maximum investigated flow (2.2 mL_gas_ s^−1^, 1.9 × 10^2^ cells mL_gas_^−1^). Total cell counts showed a similar trend, with an initial count of 9.96 × 10^5^ cells mL_gas_^−1^ at 0.07 mL_gas_ s^−1^ that declined to 6.2 × 10^3^ cells mL_gas_^−1^ at 2.20 mL_gas_ s^−1^. Cell transport per unit surface area followed the same inverse trend, ranging from 2.2 × 10^3^ MOB cm^−2^ (RSV1) to 1.0 × 10^1^ MOB cm^−2^ (SSV2) at a transported bubble surface area (*A*) of 0.8 cm^2^ s^−1^ and 39.6 cm^2^ s^−1^, respectively (Fig. 4c). The distribution pattern of the total cell counts was essentially identical, with an initial count of 9.4 ×10^4^ cells cm^−2^ at 0.8 cm^2^ s^−1^ that decreased to 3.5 × 10^2^ cells cm^−2^ at 39.6 cm^2^ s^−1^. Based on the vents per square meter at the respective study sites, the largest number of MOB was transported by IV Super Seep bubbles, where the vent density was highest, up to 700 vent vents per m^2^ (1.2 × 10^11^ cells m^−2^ d^−1^; Fig. 4d), and the lowest number at Rostocker Seep, with only 5 vents per m^2^ (7.1 × 10^8^ cells m^−2^ d^−1^) – a difference of three orders of magnitude. Note that the observed gas flow spanned over two orders of magnitude (Fig. 4).

DAPI analysis showed that the total cell abundance in the “BC control” experiments was ~3 × 10^3^ cells mL^−1^, such that mean cell counts in the “BC vent” experiments were ~17 (SS) to 94 (RS) times higher. MOB abundance in the “BC vent” was 24 (SS) to 62 (RS) times higher than in the controls (4 × 10^1^ cells mL^−1^, Supplementary Table S4). In the “BC engineered” experiments at Rostocker Seep, cell numbers of 8.0 × 10^5^ cells mL_gas_^−1^ and 4 × 10^3^ MOB cells mL_gas_^−1^ were determined, whereas at IV Super Seep only 1 × 10^4^ cells mL_gas_^−1^ and 4 × 10^2^ MOB mL_gas_^−1^ were transported into the Bubble Catcher. A comparison of the “BC engineered” and “BC vent” experiments indicated that the mean number of total transported cells in the “BC vent” experiments was two (RS) to eight (SS) times higher and four (RS) to six (SS) times higher for MOB.

### Identification of transported methanotrophs

**Figure 5:**
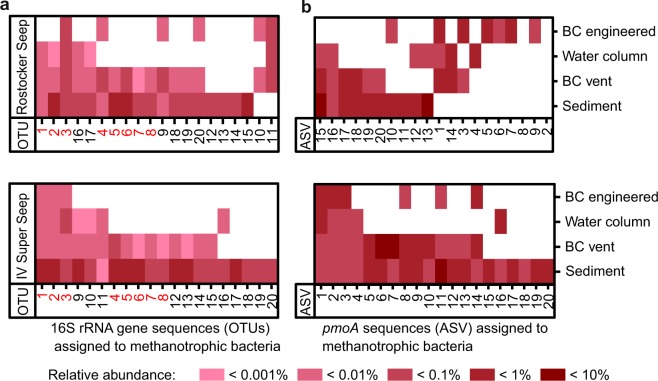
Relative abundance of (**a**) selected 16S rRNA gene sequences (OTUs) assigned to the methanotrophic family *Methylomonaceae* and (**b**) selected *pmoA* sequences (ASVs) in the different sampling groups obtained from the Rostocker Seep and IV Super Seep sites. Note that the OTUs marked in red in (**a**) had similar distribution patterns at the two seep sites. For taxonomic information on the OTUs and ASVs, see Supplementary Tables S5 and S6, respectively. Data key on figure.

To identify transported MOB, the recovered operational taxonomic units (OTUs) and amplicon sequence variants (ASVs) were searched with respect to previously known methanotrophic families. All extracted OTUs were mostly associated with unclassified methanotrophic genera (e.g., unclassified *Methylomonaceae*, Marine Methylotrophic Group 2, see Supplementary Table S5). OTUs closely related to known MOB have a high probability of being methanotrophic bacteria and hereafter referred to as methanotrophic OTUs. Twenty of 40 detected methanotrophic genera belonging to the family *Methylomonaceae* were selected for visualization (Fig. 5a). The remaining 20 OTUs were detected only in the sediment. The heat map reveals that some methanotrophic OTUs were detected in all sample groups whereas others had a divergent distribution pattern. Relative abundances based on methanotrophic reads were highest in sediment samples, lower in the “BC vent” sample, and lowest in samples taken from the “BC engineered” experiments and the water column. The highest relative abundance of single methanotrophic OTUs was 0.1–1%. In addition to the family *Methylomonaceae*, six OTUs assigned to the genus *Cycloclasticus* (Supplementary Fig. S2) were identified, including four OTUs in the sediment, “BC vent,” and water column samples. The maximum relative OTU abundance related to *Cycloclasticus* was ~1%.

From the particulate methane monooxygenase (*pmoA)* analysis, 650 ASVs were assigned, of which 42 were found in the Bubble Catcher and in sediments (Fig. 5b). The patterns for *pmoA* (Fig. 5b) and 16S rRNA genes analyses (Fig. 5a) were similar, with nine ASVs detected in all sample groups and 15 present in the sediment, “BC vent” experiments, and water columns. Of the 36 ASVs belonging to the family *Methylomonaceae*, 29 could be assigned to *Methyloglobulus morosus* and three to *Methylomicrobium kenyense*. One ASV was associated with the family *Methylocystaceae*, belonging to the order *Rhizobiales*, and four ASVs were assigned as MOB-like.

## Discussion

The high gas-venting activity in the Coal Oil Point seep field was reflected by elevated dissolved methane in the sediment and water column (Fig. 2a,d), and agreed with previous studies at similar gas-venting sites^7,29,46^. Water column methane concentrations above the COP seep sites correlated with gas seepage intensity, featuring higher methane concentrations at the more active IV Super Seep site compared to the moderately active Rostocker Seep site.

Total cell numbers in the sediment at both sites were in the range reported in previous studies conducted at COP^7,46^. MOB were detected at deeper, potentially anoxic sediment depths, a pattern that was previously described by Schmale *et al*.^7^ at Rostocker Seep, who proposed that the oxygen penetration depth is increased by bubble-driven pore water convection^46–48^. Such convection would allow MOB to settle in deeper, normally anoxic sediment strata. However, MOB cell numbers in our study were one order of magnitude lower than those reported by Schmale *et al*.^7^. This difference might be explained by the disturbance of the surface sediment by the heavy wind-induced surf in the days before the start of our fieldwork. Kersten *et al*.^49^ showed that such events have the potential to mix the upper sediment layer and to dislocate surface sediments over distance scales of meters^49,50^. In our study, this surface-sediment refreshing sand transport would have brought MOB-poor sediment into the sampling sites possibly explaining the lower cell numbers. Such an event-driven introduction of medium sand grains of similar sizes to both seep sites would explain the similarity in the bubble size distribution pattern as analyzed at all four vents (Fig. 3).

By including different seep sites, this present study allowed for a first time parameterization of the factors controlling the Bubble Transport Mechanism. These experiments showed that MOB transportation rates (i.e. cells emitted per mL of gas) were highest at a low (RS1: 0.07 mL_gas_ s^−1^, 2.3 × 10^4^ cells mL_gas_^−1^) and lowest at a high volumetric gas flow (IVSS2: 2.2 mL_gas_ s^−1^, 2 × 10^2^ cells mL_gas_^−1^) with a mean of 8 × 10^3^ cells mL_gas_^−1^ (Fig. 4), indicating that volumetric gas flow substantially affects bentho-pelagic transport efficiency. The observed decrease in transportation rates at higher gas flows implied that microorganisms were removed faster from the sediment than could be compensated by growth and/or supply from the surrounding environment. Another possible explanation for the lower transportation rate could be differences in bubble migration characteristics. At low volumetric gas flows, the sediment migration pathway collapses or partially collapses after each bubble, whereas at higher volumetric flows the conduit remains open for a prolonged period of time^51,52^. In collapsed conduits, particles, pore water, and microorganisms refill the bubble migration pathway. Thus, bubbles slowly migrating through a collapsed conduit have greater potential to interact with the surrounding sediment than do bubbles rising through an open migration path. In addition, it is likely that the continuous gas bubble sparging in an open rise path more rapidly depletes microorganisms. However, a corresponding decrease in cell numbers in the sediment taken above an active vent was not detected in our sediment samples (Fig. 2). This might have resulted from our sampling approach, in which sediment slices were homogenized before subsamples were taken for cell counting. Thus, background sediment cell numbers of 10^9^–10^10^ cells cm^−3^ could have masked localized depletion.

The overall impact of the Bubble Transport Mechanism on the abundance of MOB in the overlying water column was determined by extrapolating the numbers of cells transported per volumetric gas flow over the number of active vents per square meter of seabed. Our data show that even vents with high volumetric gas flows and accordingly low numbers of transported cells per gas bubble (e.g., IVSSV2, Fig. 4b,c) can have comparatively high cell transportation rates given the high numbers of active vents per seabed area (Fig. 4d). However, as the bubble mediated transport itself is coupled to the volumetric gas flow, we propose that factors influencing this flow, such as hydrostatic pressure^53^ (including waves and tides)^41,54^ and temperature^55^, likely affect the transportation rate of MOBs though these were beyond the scope of the data collected.

We conducted two additional types of Bubble Catcher experiments to allow for the transportation of water-borne microorganisms (“BC engineered”) and the introduction of contamination during Bubble Catcher handling (“BC control”) and “BC vent” experiments. The “BC control” experiments without gas bubble transport into the sampling cylinder featured low numbers of microorganisms, indicative of minor contamination of our samples by air- and/or water-borne microorganisms. The majority of this contamination (10^3^ cells mL^−1^) probably resulted from the scuba-diver operations with the Bubble Catcher at the seafloor. Additional microorganisms may have been introduced through sediment that was resuspended by wind and wave action^4,5^. Air-borne contamination during sample handling and filtration were likely low, due to the differences in cell concentration of air (10^3^−10^7^ cells m^−3^)^56^ and sample water (10^10^–10^11^ cells m^−3^).

Although in the “BC vent” experiments, bubbles escaping into the water column were likely loaded with bacteria, fine particles, and surfactants collected from the sediment, the “BC engineered” experiments were designed to assess the contribution of water column-borne microorganisms to the Bubble Transport Mechanism. Previous studies have shown that particles and microorganisms in the sediment adsorb to the bubble interfaces due to the surface activity of bacterial cells^24,57^. Different to the “BC vent” experiments, bubbles in the engineered bubble experiment were not pre-coated by surfactants prior to their migration through the water column. Thus, engineered bubbles likely provided a greater available surface area for transportation and therefore collected more water-column bacteria before entering the Bubble Catcher (5 cm from sediment to Bubble Catcher entrance). If the engineered bubbles were indeed more efficient in sparging microbes from the water column into the Bubble Catcher, then MOB transport rates, and total cell numbers calculated for the “BC vent” experiments would be underestimated.

The quantitatively observed transport of MOB with catalyzed reporter deposition fluorescence *in situ* hybridization (CARD-FISH) was supported by genetic analyses. Based on methanotrophic OTUs, some benthic MOB were transported from the sediment into the water column while others remained in the sediment. A bubble-mediated bentho-pelagic transport of methanotrophs was indicated by the detection of methanotrophic OTUs, in the sediment, in the “BC vent”, and in the water column samples (IV Super Seep OTUs: 1, 2, 3, 9, 10, 11; Rostocker Seep OTUs: 1, 2, 3, 17, 16). Four of these OTUs were also found in the samples from the “BC engineered” experiments (IV Super Seep: 3; Rostocker Seep: 1), which confirmed the bubble-induced stripping of bacteria from the water column into the Bubble Catcher sampling cylinder. This mechanism would have the potential to dislocate microorganisms within the water column, even across boundary layers such as pycnoclines. The detection of 18 methanotrophic OTUs in both sediment and “BC vent” samples (IV Super Seep OTUs 4–8, 12–15; Rostocker Seep OTUs 4–8, 9, 18–20), which were absent in the water column, suggests that some MOB cells transported into the Bubble Catcher either quickly sank back to the seafloor or perished in the pelagic environment. The transport patterns from 8 of 20 methanotrophic OTUs were similar at the two seep sites (red OTU numbers, Fig. 5a), whereas there was no such similarity for the ASV distribution. Microbial transport efficiency may be affected by the specific cell surface activity of microorganisms, which results, for example, from inter-species differences in outer membrane composition and extracellular components^58,59^ but also depends on the growth conditions (e.g., nutrient levels)^60^. Also, the cell position in a biofilm can influence their anchorage on particles and their likelihood of detachment by external forces^61^. In addition, a recent study on the aerosolization of bacteria and viruses at the ocean-atmosphere interface suggested that cell morphology is a critical parameter that influences bubble-induced transport between these two environments^62^.

Our *pmoA* analysis supported the 16S rRNA gene-based transportation pattern. The six ASVs found in the sediment, “BC vent” experiment, and water column provided evidence of the bubble-mediated transport of benthic MOB across these habitats. Among the transported ASVs, 37 were assigned to the family *Methylomonaceae*, which agreed with the 16S rRNA gene results. However, the identified ASVs probably belonged to unknown marine species, because the only assignments that could be made using the reference database was to a species isolated from a freshwater lake (*Methyloglobulus morosus*), whose growth is reduced already at a very low salinity^63^, as well as to a species isolated from a Kenyan soda lake, which grows optimum is at pH > 9 (*Methylomicrobium kenyense*)^64^. Based on current knowledge, neither species was likely to have existed in the sampled environment.

A previous study by Tavormina *et al*.^65^ used *pmoA* sequences from sediment and water column (3 m above sea floor) samples to investigate MOB community structure in two methane vent environments at water depths between 500 and 700 m, at the Eel River Basin and the Santa Monica Basin, respectively. A comparison of the 16 *pmoA* isolates in sediment and water samples from the two sites showed distinct methanotrophic communities with very little overlap between the two environments. In addition, the 16S rRNA gene analysis did not recover methanotrophic lineages. By contrast, we detected 40 OTUs belonging to methanotrophic lineages, of which eight were shared by the seep sediment and overlaying water column. However, using next generation sequencing methods resulted in a higher sequencing depth than achieved with the clone-library-dependent sequencing technique used by Tavormina *et al*.^65^. The difference in techniques and the respective sensitivities might explain the differences in the observed community overlap. Another possible explanation for the absence of benthic MOB in the water column in the Tavormina *et al*.^65^ study could have been the sampling depth (3 m above the seafloor). During their travel through the water column, ascending gas bubbles released at greater water depth continuously exchange gases with the surrounding water via their surfaces, resulting in a general decrease in bubble size with increasing distance from the seabed. It is expected that the constant shrinking of the gas bubbles surface during ascending^25^ increases the shear stress on the attached microbial cells leading to a preferential release of MOB at shallower water depth. For the time of our study the elevated shear stress at the bubble surface provoked by wake eddies^66^ support the detachment of microorganisms already near the seabed. Differences in environmental factors, such as oxygen concentration in the water column, seep characteristics, and volumetric gas flows, between the COP seep field and the seep sites investigated by Tavormina *et al*.^65^ may also explain the lack of MOB in the water column.

Although the 16S rRNA gene analysis indicated an overlap between the methanotrophic communities, no information could be obtained about the metabolic activity of the transported cells. The fact that some of the transported OTUs were found in the water column while others occurred only in the “BC vent” experiments suggests different survival rates of sediment-borne bacteria in the water column. However, Bubble Catcher water samples from the “BC vent” experiments provided direct evidence of methane oxidation in incubations performed over a 3-day runtime directly after Bubble Catcher subsampling. Averaged methane oxidation rates of “BC vent” (~900 nmol L^−1^ d^−1^) were three orders of magnitude higher than “BC engineered” (~0.6 nmol L^−1^ d^−1^) and five orders of magnitude higher than “BC control (~0.005 nmol L^−1^ d^−1^) rates. Thus, at least some of the transported MOB were active after their dislocation into the water column.

To assess the impact of the Bubble Transport Mechanism in the COP water column on MOB abundance, we performed a rough calculation to derive the transportation time and distance required to achieve the detected water column MOB stock (Fig. 2c). For the calculation, we assumed a constant bubble-mediated MOB input into the overlying water column and survival of the transported organisms. Calculation were performed for two different intensities of sediment-born MOB inputs: (i) maximum input as measured at the IVSS site, and (ii) minimum input as detected at the Rostocker seep site (Fig. 4). According to these assumptions, it takes about (i) 3 hours (1 km) to (ii) 14 days (121 km), respectively, of bubble-mediated transport to reach the number of water column MOB cells observed. Even if MOB cell division (about 3 days)^37^ within the methane-enriched plume water would further shorten these transportation times, our assessment shows that a relevant part of the water-column MOB stock was likely transported from gas-releasing seep sites further upstream from the COP seep field. Such a scenario is likely since the shallow waters along the southern Californian coast harbor gas-bubble releasing seep sites for at least over 100 km upcurrent^41,67^. These seeps will contribute to a high ambient water column MOB concentration while the waters are transported northwards by the coastal current system^67–69^. However, for a solid estimate of the effect of the bubble-transport mechanism on the water column MOB stock in seep regions, an isolated seep area, with low and uniform background MOB concentrations, and a defined current system would be required.

Aside from MOB, our genetic analysis further indicated the transport of OTUs assigned to the genus *Cycloclasticus* (Supplementary Fig. S2). Members of this genus have been reported to metabolize polycyclic aromatic hydrocarbons (PAHs)^70,71^ and have been playing a dominant role in the degradation of oil^72^, for example in the Deepwater Horizon oil spill^73,74^. In addition, symbiotic *Cycloclasticus* associated with mussels and sponges use short-chain alkanes rather than PAHs. However, so far no members of the genus *Cycloclasticus* were observed to oxidize methane^73,75^. We propose that *Cycloclasticus* species play a role in the degradation of petroleum compounds in the sediment and water column of COP^76,77^, with the latter being enhanced by the bubble-mediated transport. Consequently, it is conceivable that this transport mechanism influences other biogeochemical processes in the vicinity of gas seeps, too.

## Materials and Methods

### Study site

**Figure 6:**
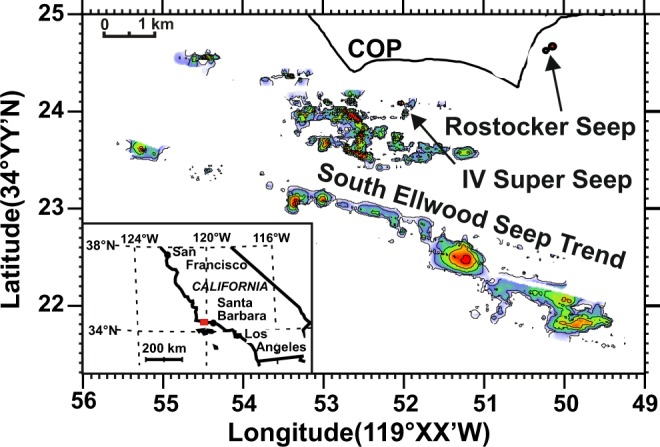
Sonar map of seep bubble intensity in the Coal Oil Point seep field. Adapted from Leifer *et al*.^78^. Matlab, Mathworks (v. R2008a) was used to create this map.

The Coal Oil Point (COP) seep in southern California, USA, extends from the coastline to 3–4 km offshore, with seepage from water depths of a few meters up to 80 m (Fig. 6)^41,78,79^. COP seeps are primarily or entirely supplied by gas, mostly methane (>90%), from the underlying Monterey Formation through faults and fractures in the overlying Sisquoc Formation^78^. Methane originates from thermogenic sources underlying the Santa Barbara Channel, at sub-seafloor depths of 3–4 km^80^. In a 1995 estimate, seabed emissions of ~1.0–1.5 × 10^5^ m^3^ d^−1^ gas (88% methane) were reported^77^. Approximately half of this methane dissolves in the water, the other half reaches the atmosphere^76^. A dissolved, submerged methane plume then is transported down-current remaining detectable for 50 km^81^.

The COP field allows perfect access for divers and equipment to a variety of gas vents that was a prerequisite for our investigations. Field experiments were conducted between 1 August and 15 September 2017 at two scuba-diver-accessible seep sites in the COP seep field (Fig. 6), chosen to span a range of seep intensities. The Rostocker Seep is located in 10-m-deep water. Its seeps have a relatively low volumetric gas flow^7^, a high methane content (>91%)^82,83^, and no oil emissions. The Isla Vista Super Seep, located at 16-m-deep water, has an intense, focused seep area surrounded by extensive dispersed small seep vents, and emits gas bubbles with >95% methane^78,83^. Previous studies in the COP seep field demonstrated methane oxidation in sediment^46^ and water column^82,84^.

### Bubble Catcher experiment and subsampling

**Figure 7:**
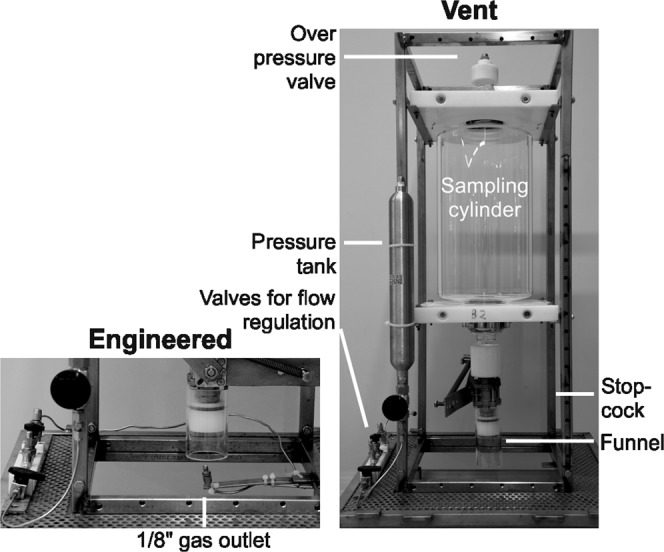
The Bubble Catcher used in the “BC vent” experiments (right) and in the “BC engineered” experiments (left).

The Bubble Catcher (outer dimensions: 35 cm/ 35 cm/ 90 cm) used herein was similar to that in Schmale *et al*.^7^, but the sampling cylinder (volume 12.7 L) was made of glass. A funnel and a stopcock directed and opened/closed the bubble flow, respectively, and a relief pressure valve prevented overpressure during surfacing (Fig. 7). A metal frame stabilized and protected the glass cylinder.

Prior to its deployment, the cylinder was filled with sterile-filtered seawater subsampled for total cells and MOB cell abundances to check for possible contamination. During the experiment, the captured bubbles (4–6 L) displaced Bubble Catcher water. Particles and microorganisms associated with the seep bubbles were released into the sterile-filtered water in the sampling cylinder after the bubbles had burst. Experiment durations and collected gas volumes were used to calculate the volumetric gas flow (mL_gas_ s^−1^). After gas collection the Bubble Catcher was returned to the boat and the BMS was deployed from a second vessel and positioned at the exact same vents. Push cores were taken last as they are disrupting the sediment. Water column sampling was conducted during the runtime of the Bubble Catcher experiments. Bubble Catcher subsampling occurred within 1 h after return to shore.

To parameterize the bubble-mediated transport process, gas bubbles were sampled from different gas vents of the Rostocker Seep and IV Super Seep sites. The sampling process is shown schematically in Supplementary Fig. S3. Three experiment types were conducted, details and an overview of the experiments duration and sampled gas volumes are provided in Table 1. For the “BC vent” experiments, divers placed the Bubble Catcher above an active natural gas vent. In the “BC engineered” experiments (“BC blank” in Schmale *et al*.^7^), the Bubble Catcher was placed above an engineered gas bubble source, created by air released from a pressure tank, positioned at least ~1 m from any gas vent. The bubble stream was adjusted to resemble volumetric flow and bubble size of natural vents in the vicinity. In “BC control” experiments, no bubbles were introduced into the Bubble Catcher, which was positioned as described for the “BC engineered” experiments. During subsampling, residual water in the Bubble Catcher was homogenized using a sterilized mixing rod, inserted through a small opening at the top of the Bubble Catcher, and filled into prepared sampling containers. The water sample was transferred via a sterilized glass funnel that was placed on the bottom of the respective container. Samples for methane oxidation rates determination and methane concentration measurement were subsampled bubble free without sample overflow, due to sample volume limitation. Total sample volume was determined by incremental filling. Methane concentration, MOB abundances, MOB community, and methane oxidation rates (only determined in the water column and Bubble Catcher) were analyzed from: (i) residual Bubble Catcher water, (ii) the sediment, and (iii) the water column.

### Sediment and water column sampling

Sediment samples were collected as push cores (length 30 cm, inner diameter 5 cm) to determine sediment methane depth profiles and confirm the presence of MOB that could be transported into the water column.

Consistent with the high vent density at IV Super Seep the cores SSV1 and SSV2 were taken from sites of active venting. Other cores were taken at a distance of ~15 cm from any active vents. Cores for methane determination were processed on land directly by subsampling sediment plugs horizontally through openings pre-drilled in the core-liner at 1.5 cm depth increments to a maximum depth of 10.5 cm, using 3 mL cut-off plastic syringes. Cores for MOB abundance analyses were cooled (4 °C) during transport to the laboratory and kept refrigerated (4 °C, 18–42 h) until subsampled in 1.5 cm increments.

The physical, chemical, and biological parameters of the water column were monitored to characterize sea water in proximity to Rostocker Seep and IV Super Seep. Salinity, temperature, and depth were monitored periodically over the course of a fieldwork day using a handheld sensor (CTD48M, Sea & Sun Technology GmbH, Germany). Water samples were collected with a 2 L handheld water sampler (LIMNOS, Finland) at depths of 1, 5, and 9 m at Rostocker Seep site and 1, 5, 10, and 15 m at IV Super Seep site.

### Methane concentration

To determine the methane concentration in the water column and Bubble Catcher, 125 mL of sample water was filled bubble-free directly into crimp vials from the Bubble Catcher and handheld water sampler, respectively. All samples were cooled (3 h, 4 °C) until processed in the lab. The crimp vials were injected with 10 µL of saturated HgCl_2_ solution/mL of sample and stored inverted^85^. The samples were analyzed for methane using the headspace method^86^, by manually injecting 30 µL of headspace gas with a gastight syringe (Hamilton 100 µL) into a gas chromatograph (Agilent GC 7890 B, temp. program 45 °C). Each sample was analyzed twice. If the mean deviation between the measurements was >1%, a third measurement was conducted. For calibration, two standard gases were used with (1) 97.7 ppm CH_4_, injection volume 50 µL, and (2) 3950 ppm CH_4_, injection volume 20 µL, in syn. air (Linde) with an uncertainty of ± 2% by manufacturer. The standards were measured five times with a mean deviation of <1% at the beginning and end of each day.

For sediment methane concentrations, 2 cm^3^ of sediment from each depth was transferred into 10 mL crimp vials filled with 5 mL of 2.5% NaOH. The vials were stored inverted at 4 °C. Methane concentrations were determined by injecting 30 µL of headspace gas from each vial as described above.

### Methane oxidation rates

Methane oxidation rates were measured in the water column and to ensure the activity of the collected MOB after Bubble Catcher sampling. 100 mL of sample water was filled bubble-free directly into crimp vials and sealed with non-toxic PTFE-coated chlorobutyl rubber stoppers (Wheaton, USA)^87^. All samples were cooled (4 °C) until further processing in the lab. Methane oxidation rates were determined according to Bussmann *et al*.^88^ with slight modifications. Thus, 20 µL of gaseous ^3^H-CH4 tracer (1:3 dilution with N_2_, ~50 kBq, specific activity 0.37–0.74 TBq/mmol, American Radiolabeled Chemicals, USA) was added to every sample. Radioactivity was measured by liquid scintillation counting (PerkinElmer, TRI-CARB 4910TR, USA). Oxidation rates were calculated using the determined rate constant multiplied by the methane concentration. For “BC vent” samples, methane saturation was assumed and calculated after Wiesenburg and colleagues^89^ because methane gas bubbles purged permanently through the sample during the experiment time.

### Quantification of MOB

MOB and total cell abundances were analyzed in sediment, water column, and Bubble Catcher samples to quantify the transport of benthic MOB into the water column. For the CARD-FISH analysis, 100 mL of Bubble Catcher sample was filled into sterile centrifuge tubes (50 ml), for water column samples the volume was adjusted to 20 ml, cooled (3 h, 4 °C) until further processing in the lab. Sediment samples were processed as described in Schmale *et al*.^7^, using the ultrasonic probe (Bandelin HD70 Sonopuls, Germany). An aliquot of 3 mL (dilution 1:3000) was filtered on 0.2 µm filters. All sample filters were prepared and analyzed according to Pernthaler *et al*.^90^. For hybridization, the probes M(γ)84 and M(γ)705, specific for type I MOB, and M(α)450, specific for type II MOB, were used as described previously^7^, except that they were pre-labeled with horseradish peroxidase. Filters were counterstained with 4′,6-diamidino-2-phenylindole (DAPI) to determine total cell counts.

Each Bubble Catcher experiment was corrected for the number of cells inside the Bubble Catcher prior to its deployment. Determined cell numbers in the “BC vent” and “BC engineered” experiments were also corrected for the cell numbers in the “BC control” experiments. The number of cells mL^−1^ was multiplied by the water volume of the residual sample to determine the number of transported cells per Bubble Catcher and then divided by the volume of captured gas to calculate the transported cells mL_gas_^−1^ (Fig. 4b). These rates were multiplied by the volumetric flow to obtain the number of transported cells s^−1^ (Fig. 4a). The number of transported cells per bubble surface area (cells cm^−2^) was determined from bubble size distributions measured by the BMS (Fig. 4c). The number of transported cells m^−2^ d^−1^ (Fig. 4d) was calculated by multiplying the number of transported cells s^−1^ by the vent density.

### DNA extraction and 16S rRNA gene/*pmoA* sequencing

For DNA analysis, Bubble Catcher and water column samples were filtered on hydrophilic polycarbonate membrane filters (0.22 µm, 47 mm, Merck Millipore, Darmstadt, Germany), which were stored at −80 °C until analysis. To ensure sufficient biomass, 0.5–1 L (water column) and 1–2 L (Bubble Catcher) sample water were filtered. For sediment samples, the sediment’s upper 1.5 cm was homogenized and a subsample was transferred to a cryogenic vial that was stored at −80 °C until extraction. DNA was extracted from one-quarter of the membrane filter (for water column samples) or 0.25 mg of wet sediment with the DNeasy PowerSoil Pro kit (Qiagen). The V3-V4 region of the 16S rRNA gene was targeted with the primer set 451f-805r (forward: CCTACGGGNGGCWGCAG, reverse: GACTACHVGGGTATCTAATCC)^91^. For *pmoA*, the primer set A189fmod and mb661rmod (forward: GGNGACYGGGAYTTCTGG, reverse: CMGGMGCAACGTCYTTACC), a combination of two established sets^65,92^, was used. LGC Genomics GmbH (Berlin, Germany) performed library preparation and sequencing on an Illumina MiSeq V3 (600 cycle, 2 × 300 bp, 5 million reads). Sequences were deposited in the European Nucleotide Archive (ENA) under accession number PRJEB34318 (16S rRNA gene) and PRJEB34319 (*pmoA*). 16S rRNA gene amplicon sequences were grouped into OTUs based on a similarity >97% and classified to the genus level.

16S rRNA gene amplicon read processing and annotation were conducted using Mothur v. 1.39.5^93^ (MiSeq SOP: accessed on 02.04.2019). Taxonomic annotation was accomplished using the Silva database (release 132), including the taxonomic changes proposed by Parks *et al*.^94^. For *pmoA* sequences, the raw sequences were processed with DADA2 v. 1.12.1^95^, which uses ASVs instead of OTUs, and taxonomically annotated according to the *pmoA* reference database^96^. The phylogenetic analysis was performed with R v. 3.5.1^97^ and phyloseq v. 1.26.0^98^.

OTUs (for 16S rRNA gene analysis) were selected based on their taxonomic relationship to described MOB. ASVs present in at least two sample groups were selected. OTUs and ASVs present in control samples were removed from the analysis. Samples were grouped regarding their sample origin (sediment, “BC vent”, water column, “BC engineered”). The mean relative abundance per group and per OTU/ASV was calculated and visualized with ggplot2 v. 3.1.0^99^.

### Bubble size distribution analysis

The bubble measurement system and analysis algorithms are described elsewhere^42,45,100–103^. Video was digitized at 60 fps. All bubbles in a sequence were identified and tracked manually due to high particle and detritus concentrations in the samples. Tracking assistance was provided by the algorithms in ImageJ v. 1.52^102^. Once a bubble was selected in two sequential frames, the algorithm predictively attempts to select the same bubble in the next frame based on the expected vertical displacement. If the predictive attempt fails, then the bubble was selected manually. The bubble equivalent spherical radius, *r*, is determined from a best-fit ellipse of the bubble outline, both with and without a convex hull. Further analysis was conducted using custom routines in MATLAB v. 2018b (Mathworks, MA). The velocity of tracked bubbles was derived and a polynomial was fit to the vertical velocity (accounting for camera tilt) as a function of *r*, *V*_*x*_(*r*). *V*_*x*_(*r*) then allowed determination of the observation frequency of bubbles of each size class in the field of view to derive a multiple count correction for the flux (number of bubbles µm^−1^ radius s^−1^), which was calculated as time-resolved. Where *V*_*x*_(*r*) varied significantly with time, an error is introduced since the algorithm uses a single *V*_*x*_(*r*) which is calculated from all bubbles measured. This error was estimated at 30% for pulsing plumes which showed the largest variation in *V*_*x*_(*r*, t).

Bubble plumes can be characterized as major or minor, where major plumes are described by a power law and are formed by bubble fragmentation, and minor plumes are described by a Gaussian function^42^. For minor plumes, the radius of the peak of the Gaussian function, termed the mode, is determined solely by sediment grain size if the emission flux (*Q*) is below a critical value^44^. For *Q* above the critical value, the mode also depends on *Q*. The characteristic mode radius was determined from a least squares linear regression fit of a Gaussian function (or functions if multiple modes) using the MATLAB curve fitting tool. Integration of the emission size distribution over *r* at each time provides the time trend in total bubble volume and surface area as well as the rates of emissions of plume bubbles *Q* (mL_gas_ s^−1^) and surface area (*A*) (cm^2^ s^−1^).

### Contribution of the Bubble Transport Mechanism to the water column MOB stock

The transportation distance (Δ*x*) needed to achieve the detected average water column MOB cell concentration (*c*) was calculated from the current velocity (*v*), the water depth (*z*), and the sediment-born MOB flux (*F*) as shown in Eq. 1.

1 $$\Delta x=\frac{c\cdot v\cdot z}{F}$$

Calculations were performed with the following variables:

**Table:** 

	*c* (cells m^−3^)	*v* (m s^−1^)	*z* (m)	*F* (cells m^−2^s^−1^)
IVSS	1 × 10^9^	0.1^69,104^	16	1.38 × 10^6^
RS	1 × 10^9^	0.1^69,104^	10	8217

## Supplementary information

Supplementary information.
