# Supplementary material for: Bubble-mediated transport of benthic microorganisms into the water column: Identification of methanotrophs and implication of seepage intensity on transport efficiency
Source: Sci Rep. 2020 Mar 13;10:4682. doi: 10.1038/s41598-020-61446-9 (PMC7070025; doi:10.1038/s41598-020-61446-9)
Supplement: Supplementary file 1 — Supplementary information. [file 41598_2020_61446_MOESM1_ESM.docx]

**Bubble-mediated transport of benthic microorganisms into the water column: Identification of methanotrophs and implication of seepage intensity on transport efficiency
Supplementary Information**

Sebastian F. A. Jordan^1^*, Tina Treude^2^, Ira Leifer^3^, René Janßen^1^, Johannes Werner^1^, Heide Schulz-Vogt^1^ and Oliver Schmale^1^*

*^1^Leibniz Institute for Baltic Sea Research Warnemünde, Rostock, Germany*

*^2^University of California, Los Angeles Department of Earth, Planetary, and Space Sciences, Los Angeles, California, USA*

*^3^Bubbleology Research International, Solvang, California, USA*

* Corresponding Authors

sebastian.jordan@io-warnemuende.de, +49 381 5197 3428

oliver.schmale@io-warnemuende.de, +49 381 5197 305

Leibniz Institute for Baltic Sea Research Warnemünde, Seestraße 15, D-18119 Rostock, Germany

Keywords: Methane, Methanotrophs, Cold seeps, Gas bubbles, Coal Oil Point

# **Supplementary Information**

**Supplementary Table S** 1. Overview of water column samples and the total and MOB counts per milliliter.

| Date | Sample | Sediment depth [cm] | Total cell counts [cells cm^−3^] | MOB [cells cm^−3^] |
| --- | --- | --- | --- | --- |
| 07.08.2017 | SSV2 | 1.5 | 1.20E+09 | 7.97E+07 |
|  |  | 3 | 9.85E+08 | 5.76E+07 |
|  |  | 4.5 | 8.66E+08 | 1.73E+07 |
|  |  | 6 | 6.65E+08 | 9.12E+06 |
|  |  | 7.5 | 7.02E+08 | 1.54E+07 |
|  |  | 9 | 8.26E+08 | 9.60E+06 |
|  |  | 10.5 | 1.14E+09 | 6.72E+06 |
| 08.08.2017 | SSV3 | 1.5 | 1.67E+09 | 6.82E+07 |
|  |  | 3 | 1.63E+09 | 1.30E+07 |
|  |  | 4.5 | 2.04E+09 | 1.97E+07 |
|  |  | 6 | 2.58E+09 | 1.01E+07 |
|  |  | 7.5 | 1.59E+09 | 1.54E+07 |
|  |  | 9 | 1.56E+09 | 5.28E+06 |
|  |  | 10.5 | 1.98E+09 | 6.24E+06 |
| 02.08.2017 | RSV3 | 1.5 | 2.65E+09 | 9.07E+07 |

**Supplementary Table S2.** Overview of water column samples and the total and MOB counts per milliliter.

| Date | Sample | Water depth [m] | Total cell counts [cells mL^−1^] | MOB [cells mL^−1^] |
| --- | --- | --- | --- | --- |
| 01.08.2017 | RS1.W1.1 | 1 | 2.43E+06 | 8.11E+04 |
| 01.08.2017 | RS1.W1.2 | 5 | 2.19E+06 | 7.13E+04 |
| 01.08.2017 | RS1.W1.3 | 9 | 2.07E+06 | 5.58E+04 |
| 02.08.2017 | RS1.W2.1 | 1 | 2.54E+06 | 8.02E+04 |
| 02.08.2017 | RS1.W2.2 | 5 | 3.26E+06 | 9.42E+04 |
| 02.08.2017 | RS1.W2.3 | 9 | 2.45E+06 | 3.95E+04 |
| 15.08.2017 | RS2.W3.1 | 1 | 1.73E+06 | 1.85E+04 |
| 15.08.2017 | RS2.W3.2 | 5 | 1.25E+06 | 1.78E+04 |
| 15.08.2017 | RS2.W3.3 | 9 | 1.76E+06 | 2.69E+04 |
| 07.08.2017 | SS1.W1.1 | 1 | 1.76E+06 | 2.86E+04 |
| 07.08.2017 | SS1.W1.2 | 5 | 1.77E+06 | 2.34E+04 |
| 07.08.2017 | SS1.W1.3 | 10 | 1.97E+06 | 2.10E+04 |
| 07.08.2017 | SS1.W1.4 | 15 | 1.16E+06 | 1.54E+04 |
| 08.08.2017 | SS1.W2.1 | 1 | 2.60E+06 | 4.46E+04 |
| 08.08.2017 | SS1.W2.2 | 5 | 3.31E+06 | 6.45E+04 |
| 08.08.2017 | SS1.W2.3 | 10 | 2.52E+06 | 3.27E+04 |
| 08.08.2017 | SS1.W2.4 | 15 | 2.85E+06 | 1.97E+04 |
| 14.08.2017 | SS2.W3.1 | 1 | 1.65E+06 | 1.88E+04 |
| 14.08.2017 | SS2.W3.2 | 5 | 1.30E+06 | 2.07E+04 |
| 14.08.2017 | SS2.W3.3 | 10 | 1.48E+06 | 1.78E+04 |
| 14.08.2017 | SS2.W3.4 | 15 | 1.32E+06 | 1.28E+04 |
| 15.09.2017 | SS3.W4.4 | 15 | 1.31E+06 | 3.45E+04 |

**Bubble Size Distribution**

The bubble emission size distribution (Φ) is described by the radius (r), peak radius (R_p_), and width (W^2^) in eq. 1 and in case of a major plume, Φ is expressed by a power law of r and with exponent (A) in eq. 2, examples of which are shown in Figure 3.

$\Phi\sim\frac{\left( r-R_{P} \right)^{2}}{W^{2}}$ (1)

$\Phi\sim r^{A}$ (2)

**Supplementary Table S3.** Overview of parameter values describing the fit equation of the bubble emission size distribution (Φ).

| Seep | Fit Number | R_P_ [µm] | W [µm] | A |
| --- | --- | --- | --- | --- |
| RSV1 | 1 | 2744 | 672 |  |
| RSV1 | 2 | 1660 | 268 |  |
| RSV2 | 3 | 1505 | 240 |  |
| RSV2 | 4 | 1709 | 380 |  |
| RSV2 | 5 |  |  | -10.7 |
| SSV1 | 6 | 580 | 118 |  |
| SSV1 | 7 | 950 | 120 |  |
| SSV1 | 8 | 1540 | 410 |  |
| SSV1 | 9 |  |  | -8.1 |
| SSV2 | 10 | 1531 | 372 |  |
| SSV2 | 11 | 2271 | 234 |  |

**Supplementary Table S4.** Overview of Bubble Catcher samples as well as total and MOB counts per milliliter.

| Date | Sample | Total cell counts [cells mL^−1^] | MOB [cells mL^−1^] |
| --- | --- | --- | --- |
| 02.08.2017 | RS1E1 | 3.76E+05 | 9.13E+02 |
| 15.08.2017 | RS1E2 | 5.65E+05 | 3.61E+03 |
| 01.08.2017 | RS1V1 | 1.08E+05 | 1.52E+03 |
| 01.08.2017 | RS1V2 | 3.14E+05 | 3.31E+03 |
| 02.08.2017 | RS1V3 | 5.16E+05 | 9.21E+03 |
| 14.08.2017 | SS1E1 | 1.21E+04 | 4.52E+02 |
| 07.08.2017 | SS1V1 | 6.39E+04 | 1.18E+03 |
| 07.08.2017 | SS1V2 | 1.63E+04 | 5.33E+02 |
| 08.08.2017 | SS1V3 | 9.32E+04 | 3.65E+03 |
| 15.09.2017 | SS3C1 | 3.69E+03 | 7.61E+01 |
| 15.09.2017 | SS3C2 | 2.99E+03 | 7.61E+01 |

**Supplementary Table S5.** Taxonomic annotation of the OTUs displayed in the heat maps in Fig. 7.

|  | OTU numeration heat map |  |  | Rostocker Seep | | | | IV Super Seep | | | |  |
| --- | --- | --- | --- | --- | --- | --- | --- | --- | --- | --- | --- | --- |
| OTU |  | Family | Genus | Sediment | RS vent | Water column | RS engineered | Sediment | BC vent | Water column | BC engineered | Control |
| 00224 | 6 | *Methylomonaceae* | Marine_Methylotrophic_Group_2 | 0.680 | 0.041 | 0 | 0 | 0.641 | 0.001 | 0 | 0 | 0 |
| 00272 | 2 |  | pItb-vmat-59 | 0.154 | 0.009 | 0.003 | 0 | 0.821 | 0.013 | 0.005 | 0.002 | 0.007 |
| 00293 | 9 |  | Marine_Methylotrophic_Group_2 | 0.004 | 0.058 | 0 | 0.008 | 0.637 | 0.009 | 0.001 | 0 | 0 |
| 00326 | 5 |  | Marine_Methylotrophic_Group_2 | 0.401 | 0.017 | 0 | 0 | 0.285 | 0.004 | 0 | 0 | 0 |
| 00334 | 13 |  | pItb-vmat-59 | 0.044 | 0 | 0 | 0 | 0.712 | 0.002 | 0 | 0 | 0 |
| 00377 | 3 |  | IheB2-23 | 0.031 | 0.148 | 0.063 | 0.045 | 0.108 | 0.017 | 0.062 | 0.006 | 0 |
| 00427 | 4 |  | pLW-20 | 0.007 | 0.059 | 0 | 0.007 | 0.308 | 0.085 | 0 | 0 | 0 |
| 00473 | 17 |  | *Methylomonaceae*_unclassified | 0.042 | 0.016 | 0.001 | 0 | 0.363 | 0 | 0 | 0 | 0 |
| 00483 | 12 |  | *Methylomonaceae*_unclassified | 0.013 | 0 | 0 | 0 | 0.394 | 0.004 | 0 | 0 | 0 |
| 00515 | 15 |  | *Methylomonaceae*_unclassified | 0.201 | 0 | 0 | 0 | 0.126 | 0.009 | 0 | 0 | 0 |
| 00542 | 1 |  | Marine_Methylotrophic_Group_2 | 0.004 | 0.020 | 0.007 | 0 | 0.234 | 0.009 | 0.007 | 0.002 | 0 |
| 00619 | 20 |  | Marine_Methylotrophic_Group_2 | 0.049 | 0.005 | 0 | 0.002 | 0.190 | 0 | 0 | 0 | 0 |
| 00622 | 11 |  | IheB2-23 | 0 | 0.067 | 0.070 | 0.021 | 0.002 | 0.007 | 0.007 | 0 | 0 |
| 00640 | 19 |  | *Methylomonaceae*_unclassified | 0.044 | 0.004 | 0 | 0 | 0.193 | 0 | 0 | 0 | 0 |
| 00808 | 16 |  | Marine_Methylotrophic_Group_2 | 0.080 | 0.014 | 0.008 | 0 | 0.030 | 0 | 0.009 | 0 | 0 |
| 00809 | 7 |  | ET-SHO | 0.015 | 0.003 | 0 | 0 | 0.143 | 0.011 | 0 | 0 | 0 |
| 00813 | 14 |  | ET-SHO | 0.018 | 0 | 0 | 0 | 0.161 | 0.004 | 0 | 0 | 0 |
| 00897 | 18 |  | IheB2-23 | 0.060 | 0.004 | 0 | 0 | 0.079 | 0 | 0 | 0 | 0 |
| 01066 | 8 |  | pItb-vmat-59 | 0.042 | 0.004 | 0 | 0 | 0.060 | 0.003 | 0 | 0 | 0 |
| 01574 | 10 |  | *Methylomonaceae*_unclassified | 0 | 0.013 | 0 | 0.004 | 0.031 | 0.004 | 0.002 | 0 | 0.008 |
| 00127 | 21 | *Cycloclasticaceae* | *Cycloclasticus* | 0.145 | 0.088 | 0.703 | 0.011 | 1.555 | 0.048 | 0.307 | 0.016 | 0.017 |
| 00229 | 22 |  | *Cycloclasticus* | 0 | 0 | 0.001 | 0 | 1.563 | 0.044 | 0.005 | 0 | 0 |
| 00230 | 23 |  | *Cycloclasticus* | 0.011 | 0 | 0 | 0 | 1.424 | 0.025 | 0.000 | 0 | 0 |
| 00609 | 24 |  | *Cycloclasticus* | 0.007 | 0.002 | 0.042 | 0 | 0.171 | 0.007 | 0.004 | 0 | 0 |
| 00657 | 25 |  | *Cycloclasticus* | 0 | 0 | 0 | 0 | 0.229 | 0.015 | 0 | 0.002 | 0 |
| 01094 | 26 |  | *Cycloclasticus* | 0.005 | 0 | 0.024 | 0.002 | 0.052 | 0.002 | 0.009 | 0 | 0 |
| 01291 | 27 |  | *Cycloclasticus* | 0 | 0 | 0 | 0 | 0.098 | 0.006 | 0 | 0 | 0 |
| 01326 | 28 |  | *Cycloclasticus* | 0 | 0 | 0 | 0 | 0.090 | 0.004 | 0 | 0 | 0 |
| 01933 | 29 |  | *Cycloclasticus* | 0 | 0 | 0 | 0 | 0.046 | 0.004 | 0 | 0 | 0 |
| 02191 | 30 |  | *Cycloclasticus* | 0 | 0 | 0 | 0 | 0.041 | 0 | 0 | 0 | 0 |
| 02752 | 31 |  | *Cycloclasticus* | 0 | 0 | 0 | 0 | 0.034 | 0 | 0 | 0 | 0 |

**Supplementary Table S6.** Taxonomic annotation of the ASVs displayed in the heat map shown in Fig. 7.

|  | ASV numeration heat map |  |  |  |  |  | IV Super Seep | | | | Rostocker Seep | | | |
| --- | --- | --- | --- | --- | --- | --- | --- | --- | --- | --- | --- | --- | --- | --- |
| ASV |  | class | order | family | genus | species | Sediment | BC vent | Water column | BC artificial | Sediment | BC vent | Water column | BC artificial |
| 00067 |  | *Gammaproteobacteria* | *Methylococcales* | *Methylomonaceae* | *Methyloglobulus* | *Methyloglobulus morosus* | 1.304 | 0.216 | 0.128 | 0.155 | 0.201 | 0.031 | 0 | 0 |
| 00038 |  | *Gammaproteobacteria* | *Methylococcales* | *Methylomonaceae* | NA | NA | 0.069 | 0.101 | 0.716 | 0.285 | 0 | 0.484 | 0.781 | 0.496 |
| 00158 |  | *Gammaproteobacteria* | *Methylococcales* | *Methylomonaceae* | *Methyloglobulus* | *Methyloglobulus morosus* | 0.396 | 0.120 | 0.078 | 0.090 | 0 | 0.225 | 0 | 0.017 |
| 00064 | 1 | *Gammaproteobacteria* | *Methylococcales* | *Methylomonaceae* | NA | NA | 0.034 | 0.095 | 0.184 | 0.212 | 0 | 0.128 | 0.137 | 0.402 |
| 00226 |  | *Gammaproteobacteria* | *Methylococcales* | *Methylomonaceae* | *Methyloglobulus* | *Methyloglobulus morosus* | 0.006 | 0.200 | 0.184 | 0.562 | 0 | 0.122 | 0 | 0 |
| 00607 | 2 | *Gammaproteobacteria* | *Methylococcales* | *Methylomonaceae* | *Methyloglobulus* | *Methyloglobulus morosus* | 0.079 | 0.106 | 0.095 | 0.130 | 0 | 0 | 0 | 0 |
| 00391 | 3 | *Gammaproteobacteria* | *Methylococcales* | *Methylomonaceae* | *Methyloglobulus* | *Methyloglobulus morosus* | 0.097 | 0.113 | 0.037 | 0.106 | 0 | 0.048 | 0 | 0.223 |
| 00195 |  | *Gammaproteobacteria* | *Methylococcales* | *Methylomonaceae* | *Methyloglobulus* | *Methyloglobulus morosus* | 0.297 | 0.444 | 0.029 | 0 | 0.360 | 0 | 0 | 0.066 |
| 00078 | 4 | *Gammaproteobacteria* | *Methylococcales* | *Methylomonaceae* | NA | NA | 0.041 | 0.045 | 0.048 | 0 | 0 | 0 | 0.158 | 0 |
| 00023 |  | *Gammaproteobacteria* | *Methylococcales* | *Methylomonaceae* | *Methyloglobulus* | *Methyloglobulus morosus* | 2.970 | 0.625 | 0 | 0 | 0.713 | 0.140 | 0 | 0.070 |
| 00017 |  | *Gammaproteobacteria* | *Methylococcales* | *Methylomonaceae* | *Methyloglobulus* | *Methyloglobulus morosus* | 3.534 | 0.144 | 0 | 0.269 | 2.084 | 0.079 | 0 | 0 |
| 00011 |  | *Gammaproteobacteria* | *Methylococcales* | *Methylomonaceae* | *Methyloglobulus* | *Methyloglobulus morosus* | 4.057 | 0.091 | 0 | 0.196 | 6.944 | 0 | 0 | 0 |
| 00133 | 5 | *Gammaproteobacteria* | *Methylococcales* | *Methylomonaceae* | *Methyloglobulus* | *Methyloglobulus morosus* | 0.215 | 1.132 | 0 | 0 | 0 | 0 | 0 | 0.138 |
| 00524 | 6 | *Gammaproteobacteria* | *Methylococcales* | *Methylomonaceae* | *Methyloglobulus* | *Methyloglobulus morosus* | 0.147 | 1.507 | 0 | 0 | 0 | 0 | 0 | 0.059 |
| 00365 | 7 | *Gammaproteobacteria* | *Methylococcales* | *Methylomonaceae* | *Methylomicrobium* | *Methylomicrobium kenyense* | 0.049 | 3.168 | 0 | 0 | 0 | 0 | 0 | 0.141 |
| 00205 | 8 | *Gammaproteobacteria* | *Methylococcales* | *Methylomonaceae* | *Methyloglobulus* | *Methyloglobulus morosus* | 0.495 | 0.191 | 0 | 0.041 | 0 | 0 | 0 | 0.141 |
| 00698 | 9 | *Alphaproteobacteria* | *Rhizobiales* | *Methylocystaceae* | NA | NA | 0.267 | 0.263 | 0 | 0 | 0 | 0 | 0 | 0.016 |
| 00715 | 10 | *Gammaproteobacteria* | *Methylococcales* | *Methylomonaceae* | *Methylomicrobium* | *Methylomicrobium kenyense* | 0.050 | 0.873 | 0 | 0 | 0.450 | 0 | 0 | 0.027 |
| 00099 | 11 | *Gammaproteobacteria* | *Methylococcales* | *Methylomonaceae* | *Methyloglobulus* | *Methyloglobulus morosus* | 1.306 | 0.018 | 0 | 0.073 | 0.312 | 0 | 0 | 0 |
| 00168 |  | *Gammaproteobacteria* | *Methylococcales* | *Methylomonaceae* | *Methyloglobulus* | *Methyloglobulus morosus* | 0.658 | 0.034 | 0 | 0 | 0 | 0.054 | 0.126 | 0 |
| 00216 |  | *Gammaproteobacteria* | *Methylococcales* | *Methylomonaceae* | *Methyloglobulus* | *Methyloglobulus morosus* | 0.049 | 0.412 | 0 | 0.171 | 0 | 0.205 | 0 | 0.031 |
| 00314 | 12 | NA | NA | NA | NA | NA | 0.370 | 0.038 | 0 | 0 | 0.651 | 0 | 0.019 | 0 |
| 00351 |  | *Gammaproteobacteria* | *Methylococcales* | *Methylomonaceae* | *Methyloglobulus* | *Methyloglobulus morosus* | 0.311 | 0.032 | 0 | 0 | 0 | 0.026 | 0.087 | 0 |
| 00175 | 13 | NA | NA | NA | NA | NA | 0.498 | 0.018 | 0 | 0 | 1.509 | 0 | 0.032 | 0 |
| 00387 | 14 | NA | NA | NA | NA | NA | 0.043 | 0.140 | 0 | 0.147 | 0 | 0.127 | 0.214 | 0 |
| 00041 |  | *Gammaproteobacteria* | *Methylococcales* | *Methylomonaceae* | *Methyloglobulus* | *Methyloglobulus morosus* | 0.764 | 0.208 | 0 | 1.247 | 2.285 | 0.662 | 0.044 | 0.046 |
| 00139 |  | *Gammaproteobacteria* | *Methylococcales* | *Methylomonaceae* | *Methyloglobulus* | *Methyloglobulus morosus* | 0.250 | 0.008 | 0.058 | 0 | 0.118 | 0.462 | 0.050 | 0.199 |
| 00255 | 15 | *Gammaproteobacteria* | *Methylococcales* | NA | NA | NA | 0.150 | 0 | 0 | 0 | 1.682 | 0.105 | 0.080 | 0 |
| 00085 |  | *Gammaproteobacteria* | *Methylococcales* | *Methylomonaceae* | *Methyloglobulus* | *Methyloglobulus morosus* | 1.065 | 0.059 | 0 | 0.155 | 0.789 | 0.323 | 0.012 | 0 |
| 00274 |  | NA | NA | NA | NA | NA | 0.029 | 0 | 0.003 | 0 | 0.180 | 0.376 | 0.019 | 0 |
| 00333 | 16 | *Gammaproteobacteria* | *Methylococcales* | *Methylomonaceae* | *Methyloglobulus* | *Methyloglobulus morosus* | 0.014 | 0 | 0.299 | 0 | 0.042 | 0.076 | 0.067 | 0 |
| 00084 |  | *Gammaproteobacteria* | *Methylococcales* | *Methylomonaceae* | NA | NA | 0.220 | 0 | 0 | 0 | 3.877 | 0.663 | 0 | 0 |
| 00052 |  | *Gammaproteobacteria* | *Methylococcales* | *Methylomonaceae* | *Methyloglobulus* | *Methyloglobulus morosus* | 1.097 | 0 | 0 | 0.065 | 2.977 | 0.476 | 0 | 0 |
| 00055 |  | *Gammaproteobacteria* | *Methylococcales* | *Methylomonaceae* | *Methyloglobulus* | *Methyloglobulus morosus* | 1.010 | 0.102 | 0 | 0.139 | 1.322 | 0.629 | 0 | 0.199 |
| 00275 |  | *Gammaproteobacteria* | *Methylococcales* | *Methylomonaceae* | *Methyloglobulus* | *Methyloglobulus morosus* | 0.056 | 0 | 0 | 0 | 0.540 | 0.493 | 0 | 0 |
| 00389 |  | *Gammaproteobacteria* | *Methylococcales* | *Methylomonaceae* | *Methylomicrobium* | *Methylomicrobium kenyense* | 0.052 | 0 | 0 | 0 | 0.948 | 0.245 | 0 | 0 |
| 00317 |  | *Gammaproteobacteria* | *Methylococcales* | *Methylomonaceae* | *Methyloglobulus* | *Methyloglobulus morosus* | 0.277 | 0 | 0 | 0 | 0.526 | 0.212 | 0 | 0 |
| 00246 | 17 | *Gammaproteobacteria* | *Methylococcales* | *Methylomonaceae* | *Methyloglobulus* | *Methyloglobulus morosus* | 0.436 | 0 | 0 | 0 | 0.582 | 0.161 | 0 | 0 |
| 00610 | 18 | *Gammaproteobacteria* | *Methylococcales* | *Methylomonaceae* | *Methyloglobulus* | *Methyloglobulus morosus* | 0.052 | 0 | 0 | 0 | 0.395 | 0.220 | 0 | 0 |
| 00376 | 19 | *Gammaproteobacteria* | *Methylococcales* | *Methylomonaceae* | *Methyloglobulus* | *Methyloglobulus morosus* | 0.264 | 0 | 0 | 0 | 0.997 | 0.023 | 0 | 0 |
| 00228 |  | *Gammaproteobacteria* | *Methylococcales* | *Methylomonaceae* | *Methyloglobulus* | *Methyloglobulus morosus* | 0.074 | 0.053 | 0 | 0.571 | 0.097 | 0.138 | 0 | 0.339 |
| 00353 | 20 | *Gammaproteobacteria* | *Methylococcales* | *Methylomonaceae* | *Methyloglobulus* | *Methyloglobulus morosus* | 0.430 | 0 | 0 | 0 | 0.201 | 0.061 | 0 | 0 |


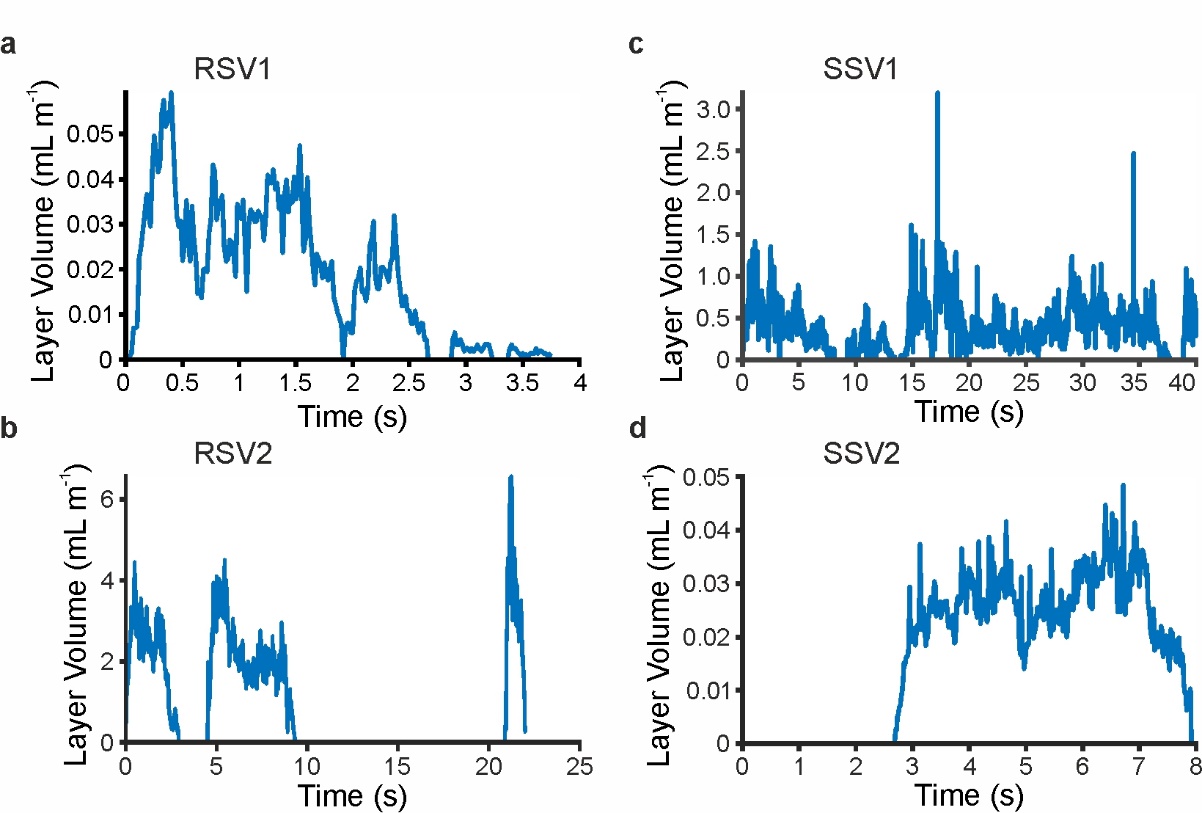


**Supplementary Figure S1.** Layer volume flux (the bubble volume in a 1-cm-thick layer) for the four Bubble Catcher and BMS studied vents: (a) and (b) Rostocker Seep, (c) and (d) IV Super Seep. Vent name labeled on figure.


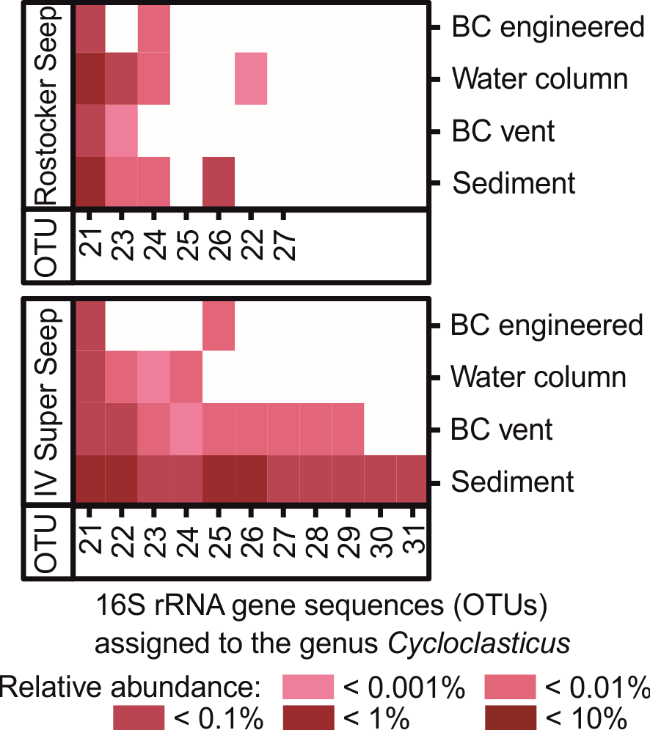


**Supplementary Figure S2.** Heat map showing the abundances of OTUs belonging to the genus *Cycloclasticus* as detected in the different habitats.


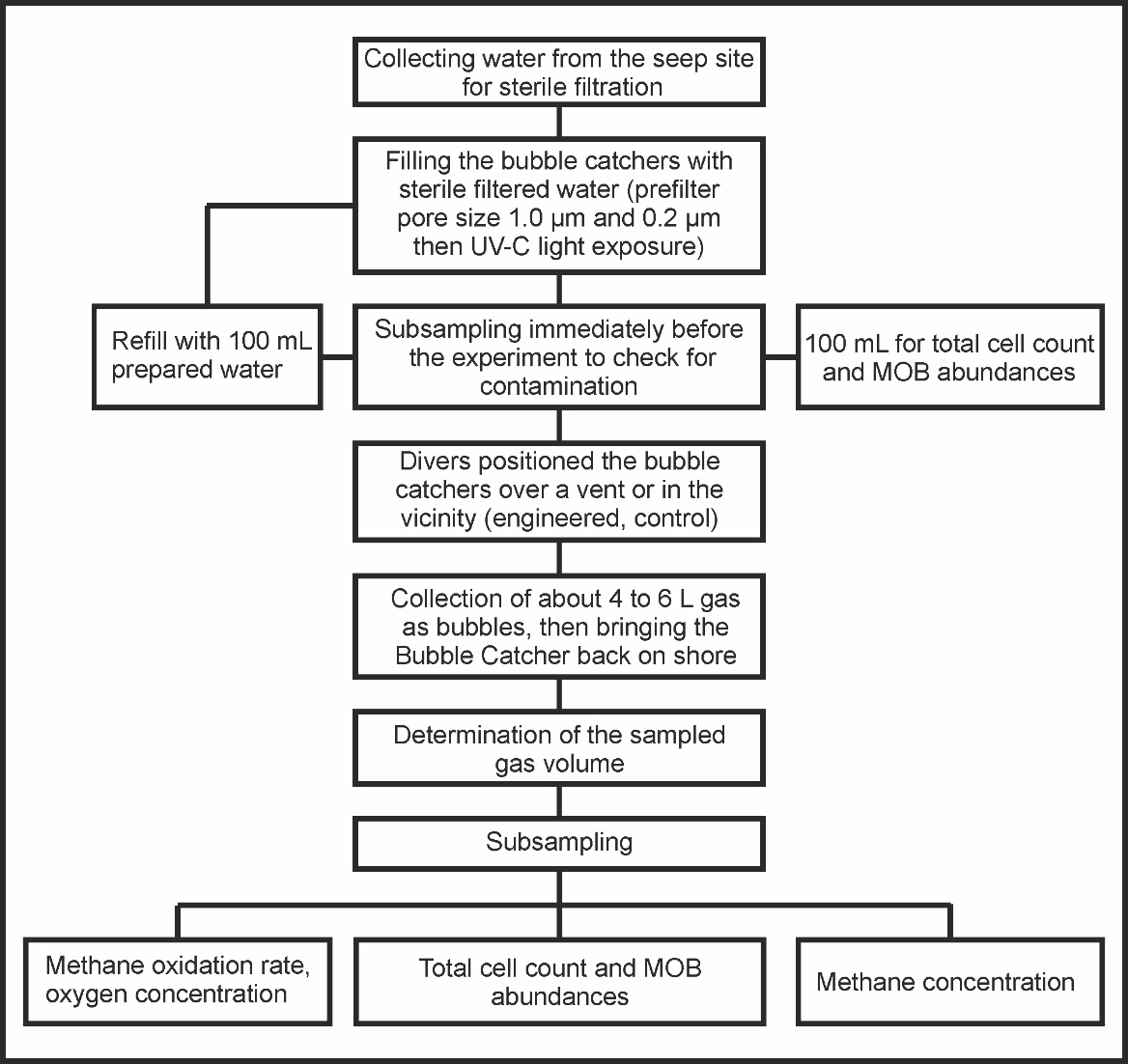


**Supplementary Figure S3.** Flow chart of the Bubble Catcher experiment procedure and subsampling. For further details, see Section 2.2.1.
